# The Role of Preoperative Computed Tomography on the Quality of Reduction and Outcomes in Intertrochanteric Fracture: A Controlled Trial

**DOI:** 10.1155/2021/8854292

**Published:** 2021-02-12

**Authors:** Tao Ma, Lin-Jie Hao, Peng-Fei Wen, Ya-Kang Wang, Hu Wang, Bin-Fei Zhang, Yu-Min Zhang

**Affiliations:** ^1^Department of Joint Surgery, Honghui Hospital, Xi'an Jiaotong University, Beilin District, Xi'an, Shaanxi Province, China; ^2^Department of Orthopedic Trauma, Honghui Hospital, Xi'an Jiaotong University, Beilin District, Xi'an, Shaanxi Province, China

## Abstract

**Purpose:**

The study is aimed at assessing the role of preoperative computerised tomography (CT) examination in the quality of reduction and outcomes in elderly patients with intertrochanteric fracture.

**Methods:**

The elderly patients with an intertrochanteric fracture who were treated with proximal femoral nail antirotation were included. The patients were divided into the CT group and the no-CT group according to the presence of preoperative CT examination. Patients' baseline characteristics, quality of reduction, and function were recorded at follow-up. Functional outcomes were evaluated using the Harris hip scores (HHS).

**Results:**

Totally, the study included 182 patients with intertrochanteric fractures, with 85 in the CT group and 97 in the no-CT group, admitted between January 2018 and June 2019. There was no difference in the quality of reduction, HHS, the fracture healing, or postoperative complications between the CT group and the no-CT group. However, the CT group experienced the shorter mean operative time and blood transfusion, compared to the no-CT group.

**Conclusions:**

The preoperative CT examination seems to be excessive for elderly patients with an intertrochanteric fracture.

## 1. Introduction

Intertrochanteric fracture is the extracapsular fracture of the proximal femur between the greater and lesser trochanters, which often suffered low energy falls in osteoporotic patients. The incidence rates per 100,000 for a primary diagnosis of intertrochanteric fractures were 171 [1] and have been kept increasing recently. These fractures accounted for an annual estimate of $52,512 per patient in America and representing 44% of all hip fracture costs [1].

Operative treatment is the main strategy to reduce the complications in bedding and improve the prognosis. Usually, sliding hip compression screw [2] and intramedullary nail [3] are the efficacy implants, and the fixing strategy by intramedullary nail is more and more popular currently, especially proximal femoral nail antirotation (PFNA) [3]. Duration of the operation, it is essential that high quality of reduction could be achieved [4]. Good reduction could provide enough stability and reduce the postoperative complications [5, 6] and improve the outcomes of intertrochanteric fractures [7].

Therefore, it is necessary to carefully evaluate the fracture displacement and angle before operation. However, the information of displacement provided by X-ray films is usually limited, and preoperative three-dimensional (3D) computed tomography (CT) reconstruction imaging could illustrate the displacement directly. The study from Shoda et al. has found that it is difficult to evaluate fracture patterns involving the greater trochanter, especially large oblique fragments including the lesser trochanter, using X-ray plains, and the 3D-CT shows the fracture line very clearly, making it easy to classify the fracture pattern [8]. Li and Lin recommend that CT could reduce fixation failure of intramedullary nailing for unstable type of intertrochanteric fractures in a comment [9]. In addition, it is reported that a new classification by CT could describe fracture morphology and predict the possibility of achieving stable reduction and the risk of complications following intramedullary fixation [10].

However, whether the quality of reduction could be improved through adding the preoperative CT examination, there is no identified conclusion. Based on the advantages reported above, this study is aimed at proving the hypothesis: preoperative CT examination could improve the quality of fracture reduction and outcomes. Therefore, we conducted a retrospective analysis to better verify this hypothesis.

## 2. Materials and Methods

### 2.1. Patient Selection

The inclusion criteria were (1) age ≥ 65 years; (2) history of falling from height, slipping, traffic accident, or other; (3) hip pain, tenderness, dysfunction, ecchymosis, and local swelling; (4) unilateral intertrochanteric fractures were confirmed using radiography; (5) operative treatment of closed reduction and internal fixation was undergone; (6) the PFNA was used to fix the fractures; and (7) at least 6 months of follow-up.

The exclusion criteria were (1) age < 65 years, (2) multiple injuries, (3) serious comorbidities and could not suffer from the operation, and (4) the patients received the dynamic hip screws.

We searched the medical system records for patients with intertrochanteric fractures who were surgically treated with PFNA. The patient record search period was from January 2018 to June 2019. All patients were admitted to a level I trauma center in Xi'an, China, and the surgery was conducted by a team of two chief physicians and four attending physicians that treated more than 200 intertrochanteric fractures with PFNA annually.

### 2.2. The Primary Examination for the Patients

When a patient arrived at the department of emergency, the physicians and surgeons would have a physical examination and give the examination items of X-rays and others. Particularly, whether needing of CT scan or not depended on doctors (Y-L L, L-L H, and H G). Upon admission of patients with intertrochanteric fractures, the blood routine test and other blood sample were examined to assess the hidden blood loss. If needed, fluid and blood transfusions were administered immediately.

### 2.3. Surgical Strategy and Technique

For patients in a stable condition, the operation was performed as soon as possible, most between days 1 and 3 postinjury. All operations were performed under general anesthesia by the same team.

After placing the patient on the traction table, the closed reduction was tried to perform by tracting the injured lower extremity and internal/external rotation. Throughout the operation, the C-arm fluoroscopy was used to check the reduction and the procedure of inserting the nail. The anterior-posterior and lateral views of the hip were used to assess the quality of reduction. If the quality is poor, there would be another reduction. If needed, a small incision is assisted to reduction with periosteal elevator or 90-degree pliers. Once the reduction was acceptable, it was the time to insert the nail.

The guide needle was used to locate the point of insertion by puncturing the skin, from the skin about 5 cm above the tip of the greater trochanter. Adjusting the guide needle, to ensure the guide needle was at slight medial of the tip of the greater trochanter on anterior-posterior view, at the middle of the proximal femur on lateral view. After determining the position of the guide needle, a 5 cm incision along the direction of the guide needle was cut. The subcutaneous tissue, superficial fascia, and deep fascia were cut sequentially, and the lateral muscles were split longitudinally to the tip of the greater trochanter. After reconfirming the fracture reduction, the guide needle was inserted into the proximal femur, and the position of the needle on the anterior-posterior and lateral view was checked. The proximal femur was gradually reamed at a faster rate and forwards slowly, and then, a 170 mm or longer nail was implanted (Tianjin Zhengtian Medical Instrument Co., Ltd., Tianjin, China). After adjusting the height of the nail, the head screw was fixed. It is noticed that the position of the guide needle under C-arm fluoroscopy was opportune so that the guide needle in the center of the femoral head in the anterior-posterior and lateral position was the smallest. After measuring the length of the head screw and reaming, the corresponding head screw was implanted and the fracture was compressed. Then, a distal locking screw was implanted under the directing frame, and a tail screw was locked. After washing out the incision, bleeding was assessed. No drainage tube was placed for all patients.

### 2.4. Follow-Up

After operative 4-5 days, the patients would be discharged from the hospital according to the recovery situation. The patients were recommended to perform isometric exercises and joint exercises in bed as soon as possible. Each patient was required to undergo partial weight-bearing at 2-4 weeks postoperatively. The timing of full weight-bearing was determined according to fracture healing. The patients would be asked to the outpatient department at least once per month for the first 6 months postoperatively, and X-ray was used to evaluate the fracture union status. If the difficulty in fracture healing was found at 6 months postoperatively, we usually changed the follow-up to every month.

### 2.5. Outcomes

The primary outcomes were quality of reduction and HHS. The secondary outcomes were operative time, intraoperative blood loss, blood transfusion, intraoperative liquid, follow-up time, weight-bearing time, clinical healing time, and complications (deep vein thrombosis, wound infection, revision, and mortality). Chang reduction quality criteria were used as the tool to assess the quality of reduction [4, 11], based on the concepts of positive medial cortical support and negative medial cortical support.

### 2.6. Statistical Analysis

Statistical analysis was performed using SPSS version 25.0 (SPSS Inc., Chicago, IL, USA). We assessed whether measurement data were normally distributed using the Shapiro-Wilk test. We then analysed the data using independent-samples *t*-tests. For frequency data, the chi-square or Fisher's exact test was used. Differences were considered statistically significant if *P* was <0.05.

## 3. Results

### 3.1. Clinical Characteristics

This study included 182 patients with intertrochanteric fractures. The three doctors (Y-L L, L-L H, and H G) from the department of emergency were given the CT/no-CT for intertrochanteric fractures 30/31, 26/34, and 29/32, respectively; there were no significant differences between these doctors (chi‐square = 0.441, *P* = 0.802).

Totally, the CT group included 85 patients, and the no-CT group included 97 patients, admitted between January 2018 and June 2019. All 182 operations were performed by the same team. The mean age was 80.35 years in the CT group and 79.37 years in the no-CT group, respectively. There were 126 females and 56 males. Females accounted for 75.30% and 63.92% of patients in the CT group and the no-CT group, without the significant differences in sex distribution (*P* = 0.097). Mechanisms of injury included slipped, high falling, accident, and other. The most common mechanism was slipped, which occurred in 89.41% and 85.57% of patients in the CT group and the no-CT group, respectively. Preoperative visual analogue scale (VAS) scores ranged from 1 to 5, and it was 3.78 ± 0.97 and 3.89 ± 0.84 in the CT group and the no-CT group, respectively.

The intertrochanteric fractures were divided into 9 subgroups; there were 20, 60, and 5 of the CT group and 35, 55, and 7 of the no-CT group in 31A1, 31A2, and 31A3 subgroups, respectively. We found no significant difference in fracture types between the two groups (*P* = 0.186).

In addition, we found there were no significant differences in the comorbidities (hypertension, diabetes, stroke, and associated injuries). The time from injury to admission was 0.96 ± 0.80 days and 1.03 ± 0.90 days in the CT group and the no-CT group, respectively. The time from admission to operation was 2.38 ± 0.83 days and 2.40 ± 0.92 days in the CT group and the no-CT group, respectively. The length of stay in hospital in the CT group (6.29 ± 2.37 days) was similar to that in the no-CT group (6.45 ± 1.63 days). Detailed baseline information is shown in Table 1.

### 3.2. Comparison of Operative Characteristics

Mean operative times were 56.94 ± 17.45 mins and 63.48 ± 14.08 mins in the CT and no-CT groups, respectively. The operative times in the CT group were shorter than those in the no-CT group (*P* = 0.006). In addition, the blood transfusion in the CT group (1.08 ± 1.14 U) was less than that in the no-CT group (1.44 ± 1.11 U, *P* = 0.035). The intraoperative blood loss was 65.88 ± 18.85 ml in the CT group and 70.62 ± 25.97 ml in the no-CT group, without significant differences. Also, there were no significant differences in the intraoperative liquid between the two groups (*P* = 0.351), CT group (1276.47 ± 282.27 ml) versus no-CT group (1237.11 ± 284.43; Table 2).

### 3.3. Follow-Up and Fracture Healing

Follow-up time was not significantly different between the two groups (11.25 ± 3.95 months in the CT group and 10.10 ± 4.22 months in the no-CT group; *P* = 0.062). During the follow-up, time to postoperative weight-bearing and time to clinical healing were assessed. The partial weight-bearing with crutches was allowed at 2.62 ± 0.91 and 2.65 ± 0.98 weeks after the operation in the CT and no-CT groups, respectively. We evaluated clinical healing on the basis of radiographic findings, symptoms, and signs. Time to clinical healing was 5.87 ± 1.11 and 5.60 ± 0.99 months in the CT and no-CT groups, respectively. We found there were no significant differences between the two groups in the time to weight-bearing or clinical healing (Table 2).

### 3.4. Functional Outcomes

Mean HHS at final follow-up were 83.85 ± 11.74 in the CT group and 84.93 ± 8.76 in the no-CT group, with no significant difference (*P* = 0.479; Table 2). In the 31A1 subgroup, mean HHS were 86.70 ± 3.63 in the CT group and 82.26 ± 13.03 in the no-CT group (*P* = 0.143). In the 31A2 subgroup, mean HHS were 82.97 ± 13.17 in the CT group and 86.44 ± 4.46 in the no-CT group (*P* = 0.051). In the 31A3 subgroup, mean HHS were 86.60 ± 3.51 in the CT group and 86.14 ± 3.85 in the no-CT group (*P* = 0.838).

### 3.5. Quality of Reduction

The quality of reduction was assessed after the operation by the X-ray films. The quality was divided into three levels: excellent, acceptable, and poor. In the CT group, the excellent level took 56% of all and the acceptable level took 42% of all. In the no-CT group, the excellent level took 60% of all and the acceptable level took 36% of all. There were no significant statistical differences in the distribution (*P* = 0.593; Table 2). The 6 patients who suffered a poor reduction were classified into 31A2.2, 31A2.3, and 31A3 types.

### 3.6. Postoperative Complications

Deep vein thrombosis, superficial infection, revision, and mortality were assessed after the operation (Table 2). No deaths occurred during the hospital stay, and five deaths occurred during the follow-up, three patients in the CT group and two patients in the no-CT group. The reason of death was cardiovascular and cerebrovascular disease. In the CT and no-CT groups, 35 and 27 patients developed into deep vein thrombosis, respectively, with no significant difference in frequency (*P* = 0.058). In the CT group, one patient had superficial infection. In the no-CT group, two patients had superficial infection. All 3 patients were treated with antibiotics and wound care, and the infections ultimately healed. No significant difference in infection rate was found between the two groups. None of the patients experienced fixation failure needing revision during follow-up.

## 4. Discussion

In the X-ray view, the AO/OTA classification [12, 13] and the stability of intertrochanteric fracture could be identified. Also, the implants designed for intramedullary or extramedullary fixation could be chosen. However, these “classic” X-rays based classification systems are thought to be insufficient and have limited ability to provide detailed explanations and accurate information about the actual fracture morphology, especially those involving large oblique fragments that include the lesser trochanter, and with coronal fragment [14–16]. With advances in radiography, CT and 3D-CT have been widely used in clinical settings to provide precise evaluation and diagnosis in orthopedics. CT/3D CT examination was more reliable and more helpful for preoperative assessment, especially for the performance of an intramedullary fixation [14]. There were indications that there was benefit for the use of CT, especially for fractures considered unstable [17].

As for CT using on intertrochanteric fractures, most of the studies were focusing on clustering of morphological fracture lines. Li et al. have proposed the 3D model classification can be used to describe fracture morphology and predict the possibility of achieving stable reduction [10]. Sharma et al. found that there were some important differences between the 3D-CT appearances and AO classification of intertrochanteric fractures [18]. Futamura et al. have built a new classification focusing on the relationship between the attachment of the iliofemoral ligament and the course of the fracture line for intertrochanteric fractures [19]. However, there was no study relationship between CT and clinical results.

In this controlled study, we aimed to prove whether preoperative CT examination could improve the quality of fracture reduction and outcomes or not. Our results showed there were no differences in the quality of reduction or HHS between the CT group and the no-CT group. Also, the fracture healing or postoperative complications did not show the differences in the two groups. However, the CT group experienced shorter mean operative time and blood transfusion, compared to the no-CT group.

The groups in this study were divided as absence or presence of preoperative CT. Our emergency department was mainly responsible for triage of patients, and three doctors (Y-L L, L-L H, and H G) prescribed CT examination of hips based on the thought mainly to clarify the scope of the fracture. The chi-square test result showed that there was no difference in the distribution of CT and no-CT among these three doctors. Through our return visits, we have found that they did not know the classification of intertrochanteric fractures and did not care about the integrity of the lateral wall of the proximal femur, and there was no preference on prescribing CT examinations. In addition, from Table 1, we could find that the baseline information of CT patients and no-CT patients was comparable, especially in the classification of fracture. The above situation explains that the possibility of selective bias was small.

We found there was no difference in the quality of reduction between the CT and no-CT groups. The result is converse to our initial hypothesis, and preoperative CT examination may not improve the quality of fracture reduction. In our study, the quality of excellent and acceptable level takes 98% of all patients in the CT group and takes 96% of all patients in the no-CT group. It seems that CT examination has no positive influence on the final outcomes and X-rays could provide enough information about fracture morphology for operation. In 2016, the study from van Embden et al. confirmed that intertrochanteric fractures can be reliably classified on both radiographs and CT, according to the main groups of the AO classification. Thus, the author thought that the clinical relevance of CT for classification of trochanteric fractures seems low [20]. The result from this study corresponds to the author's opinion. In the field of HHS, the CT examination is also shown the excess role on outcomes.

In this study, the operative time and blood transfusion in the CT group are shorter than the no-CT group. The one possible reason is that the 3D-CT provides an intuitive morphological map of fracture and a stable or unstable fracture is identified by 3D-CT; the process of reduction is relatively easy to achieve. When we compare the quality of reduction, HHS, operative time, and blood transfusion, in 31A1, 31A2, and 31A3 fracture subgroup, respectively, there were no differences in the CT and no-CT groups, but the operative time in the CT group (55.83 ± 16.92) was shorter than that in the no-CT group (64.67 ± 13.64) in 31A2 subgroup (*t* = 3.096, *P* = 0.002). The reason is that the patients in the 31A2 subgroup take 70% (60/85) of the CT group and 57% (55/97) of the no-CT group.

It is noticed that not all of the patients received operation within 48 hours after injury, although many guidelines point out that exceeding 48 hours was associated with increased mortality [21]. Because many patients in China have more serious comorbidities [22–24], these medical diseases were not properly controlled before the injury and would further develop or aggravate after the injury, due to the controlling these medical diseases, making some operations more than 48 hours. However, a recent high-quality study showed that accelerated surgery (surgery within 6 h of diagnosis) did not significantly lower the risk of mortality or a composite of major complications compared with standard care [25]. Therefore, we are not always pursuing early surgery, but to reduce perioperative risks and improve prognosis, through more individualized diagnosis and treatment.

Certainly, there are some aspects of limitation in this study. Firstly, the patients in this study were not divided into two groups randomly. It was based on absence or presence of preoperative CT prescribed by the doctors from the department of emergency. Although there was no preference on prescribing CT examinations and the baseline is comparable, there is the chance of introducing patients' selection bias. Secondly, the quality of reduction is assessed as the criterion from Chang et al. [4]. So far, it is not widely used concept of positive medial cortical support or negative medial cortical support or neutral position to assess the quality of reduction.

## 5. Conclusions

In conclusion, preoperative CT examination might not improve the fracture reduction quality and outcomes but might reduce operative time and the blood transfusion. The preoperative CT examination seems to be excessive for elderly patients with intertrochanteric fractures.

## Figures and Tables

**Table 1:** The baseline characteristics in the CT group and the no-CT group.

	CT group	No-CT group	*t*/chi-square	*P*
No. of patients	85	97		
Age	80.35 ± 7.18	79.37 ± 7.41	-0.905	0.367
Sex				
Female	64	62	2.753	0.097
Male	21	35		
Mechanism of injury				
Slipped	76	83	0.730	0.866
High falling	2	4		
Accident	3	4		
Other	4	6		
Preoperative VAS	3.78 ± 0.97	3.89 ± 0.84	0.822	0.412
AO/OTA classification				
A1.1	1	2		
A1.2	6	15		
A1.3	13	18		
A2.1	11	13		
A2.2	30	15	11.289	0.186
A2.3	19	27		
A3.1	1	2		
A3.2	3	4		
A3.3	1	1		
Comorbidities				
Hypertension	44	53	0.150	0.698
Diabetes	32	38	0.045	0.833
Stroke	20	33	2.416	0.120
Associated injuries	8	7	0.289	0.591
Days from injury to admission (days)	0.96 ± 0.80	1.03 ± 0.90	0.525	0.600
Days from admission to operation (days)	2.38 ± 0.83	2.40 ± 0.92	0.196	0.845
Length of stay in hospital (days)	6.29 ± 2.37	6.45 ± 1.63	0.522	0.602

**Table 2:** The primary and secondary outcomes between the two groups.

	CT group (*n* = 85)	No-CT group (*n* = 97)	*t*/chi-square	*P*
Operative time (mins)	56.94 ± 17.45	63.48 ± 14.08	2.798	0.006
Intraoperative blood loss (ml)	65.88 ± 18.85	70.62 ± 25.97	1.419	0.158
Blood transfusion (U)	1.08 ± 1.14	1.44 ± 1.11	2.122	0.035
Intraoperative liquid (ml)	1276.47 ± 282.27	1237.11 ± 284.43	-0.935	0.351
Follow-up time (months)	11.25 ± 3.95	10.10 ± 4.22	-1.881	0.062
Weight-bearing time (weeks)	2.62 ± 0.91	2.65 ± 0.98	0.184	0.854
Clinical healing time (months)	5.87 ± 1.11	5.60 ± 0.99	-1.755	0.081
Complications				
Deep vein thrombosis	35	27	3.590	0.058
Superficial infection	1	2	0.000	1.000
Revision	0	0	—	—
Mortality	3	2	0.022	0.881
HHS scores	83.85 ± 11.74	84.93 ± 8.76	0.709	0.479
Quality of reduction				
Excellent (%)	47 (56)	58 (60)	1.046	0.593
Acceptable (%)	36 (42)	35 (36)		
Poor (%)	2 (2)	4 (4)		

## Data Availability

The survey was implemented by Xi'an Honghui Hospital. According to relevant regulations, the data could not be shared.

## Abbreviations

CT: Computed tomography
3D-CT: Three-dimensional computerised tomography
HHS: Harris hip scores
PFNA: Proximal femoral nail antirotation.
